# Network meta-analysis of targeted therapies for diffuse large B cell lymphoma

**DOI:** 10.1186/s12885-020-07715-2

**Published:** 2020-12-11

**Authors:** Jie Wang, Jun Huang, Qing Zeng

**Affiliations:** 1 Department of Hematology, West China Hospital of Sichuan University, No. 37 Guoxuexiang Street, Chengdu, 610041 Sichuan China; 2 West China Medical School, Sichuan University, Chengdu, Sichuan China

**Keywords:** Targeted therapy, DLBCL, Network meta-analysis

## Abstract

**Background:**

The purpose of this network meta-analysis of randomized controlled trials (RCTs) was to compare rank targeted therapies for patients with diffuse large B-cell lymphoma (DLBCL).

**Methods:**

The PubMed, EmBase, and Cochrane library electronic databases were systematically searched throughout December 2019. Direct and indirect evidence from relevant RCTs was identified for network meta-analysis. The pooled results for grade 3 or greater adverse events between targeted therapies and chemotherapy were calculated using a random-effects model.

**Results:**

A total of 18 RCTs enrolling 8207 DLBCL patients were selected for the final meta-analysis. The results of the network analysis indicated that the addition of dacetuzumab (74.8%) to rituximab-based regimens or lenalidomide (77.1%) was associated with better therapeutic effects on overall survival, whereas dacetuzumab (80.4%) or bortezomib (70.8%) added to rituximab was most likely to improve events-free survival. Moreover, lenalidomide (93.8%) and I-tositumomab (77.2%) were associated with higher overall response rates. Finally, patients receiving targeted therapies were associated with an increased risk of diarrhea (RR: 2.63; 95%CI: 1.18–5.86; *P* = 0.019), and thrombocytopenia (RR: 1.41; 95%CI: 1.05–1.90; *P* = 0.023).

**Conclusions:**

This study provides the best treatment strategy for DLBCL patients in terms of overall survival, events-free survival, and overall response rate. The findings of this study require validation with further large-scale RCTs.

**Supplementary Information:**

The online version contains supplementary material available at 10.1186/s12885-020-07715-2.

## Background

Diffuse large B-cell lymphoma (DLBCL) is the most common lymphoma in adults, accounting for nearly 30–35% of malignancy in all newly diagnosed B-cell lymphomas. It is characteristically aggressive and potentially curable [1]. Diffuse large B-cell lymphoma is heterogeneous in morphology, genetics, and clinical behavior, and its outcomes can be predicted by several prognostic scores [2–4]. Two major subtypes of DLBCL, germinal center B-cell-like (GCB) and activated B-cell-like (ABC), account for approximately 50 and 40% of DLBCL diagnoses, respectively [5–7]. However, the remaining 10–15% of DLBCL patients do not meet the criteria of either the GCB or the ABC subtype, and, combined with ABC DLBCL patients, can be regarded as non-GCB DLBCL patients [8].

Today, the addition of the monoclonal CD20 antibody rituximab to primary treatment regimens has greatly improved outcomes for DLBCL patients [9–11]. The long-term cure rate after rituximab-containing conventional chemotherapy regimens is > 80.0% in young patients with good prognoses [10]. Moreover, the prognoses for patients at intermediate to high risk according to the International Prognostic Index are also improved by similar chemoimmunotherapy regimens, whereas the therapeutic effects remain unsatisfactory for residual relapse risk patients [9]. Several other targeted therapies have already been introduced for DLBCL patients at various stages, but evidence on the therapeutic effects of these agents on the prognosis of DLBCL is both limited and inconclusive. Therefore, we attempted a large-scale examination of the available evidence to evaluate the best treatment option for DLBCL patients, and summarized the direct and indirect evidence comparing different agents using a network meta-analysis approach.

## Methods

### Search strategy and selection criteria

This meta-analysis was performed according to Preferred Reporting Items for Systematic Reviews and Meta-Analyses (PRISMA) methodology [12]. Throughout December 2019, we systematically searched the PubMed, EmBase, and Cochrane Central Register of Controlled trials databases with the following keywords: (“Diffuse large B cell lymphoma” OR “DLBCL”) AND (“random” or “blind”). We also conducted manual searches of reference lists from all the relevant original and review articles to identify additional eligible studies.

The literature search and study selection were independently carried out by two authors, and any disagreement was resolved by a third author. Studies were included if the following inclusion criteria were met: (1) Patients: all patients were diagnosed with DLBCL; (2) Intervention: rituximab-, I-tositumomab-, bevacizumab-, bortezomib-, dacetuzumab-, ibrutinib-, ofatumumab-, obinutuzumab-, or lenalidomide-based treatment regimens were used; (3) Control: chemotherapy or rituximab-based chemotherapy was used as a control; (4) Outcomes: the primary outcomes were overall survival (OS), events-free survival (EFS), and overall response rate (ORR), while the secondary outcomes included any potential adverse events; and (5) Study design: all included studies had to have a randomized controlled trial (RCT) design. Exclusion criteria included basic studies and genotype-related studies. Further, reviews, editorials, letters, and conference papers without sufficient data were excluded.

### Data collection and quality assessment

The following items were extracted from each study: first authors’ surname, publication year, country, sample size, mean age, number of men and women, disease status, stage, intervention, chemotherapy regimen, and reported outcomes. The methodological quality of the included studies was assessed using the JADAD scale, which is based on the following five items: randomization, concealment of the treatment allocation, blinding, completeness of follow-up, and the use of intention-to-treat analysis [13]. Data extraction and quality assessment were conducted independently by two authors. Information was examined and adjudicated independently by an additional author referring to the original studies.

### Statistical analyses

A network meta-analysis was conducted for indirect and mixed comparisons of various agents [14]. The loop-specific approach, which assesses the difference between direct and indirect estimates for a specific comparison in the loop, was employed to check for the presence of inconsistency [15]. A design-by treatment interaction inconsistency model was used to check the assumption of consistency across the entire network [14]. After this, an inconsistent model was employed due to the potential heterogeneity among included patients. The surface under the cumulative ranking (SUCRA) probabilities were calculated to rank the treatments for each outcome [16]. Publication biases for primary outcomes were calculated using comparison-adjusted funnel plots [17]. Moreover, the pooled results for potential grade 3 or greater adverse events were calculated using the relative risk (RR) with corresponding 95% confidence intervals (CI) using a random-effects model [18, 19]. The potential impacts of disease status on the prognosis of DLBCL were also illustrated by subgroup analysis. Heterogeneity across included trials was calculated using the I^2^ and Q statistics, and *P* < 0.10 was considered as significant heterogeneity [20, 21]. A two-side *p* value < 0.05 was considered statistically significant for all analysis. All statistical analyses were performed using STATA (Version 10.0, Stata Corp., College Station, TX, USA).

## Results

### Literature search

**Fig. 1:**
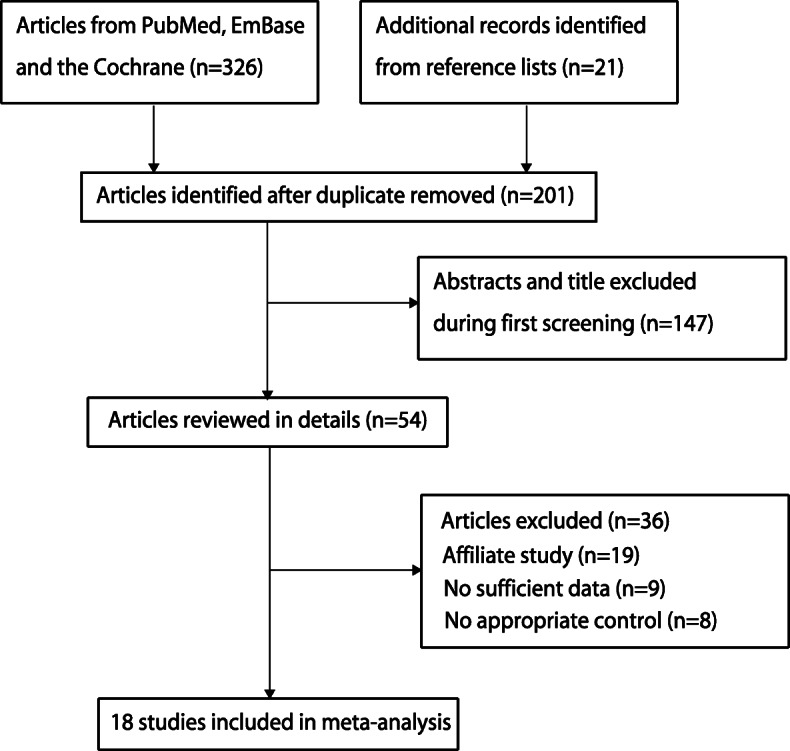
PRISMA flowchart illustrating the selection of studies included in our analyses

In our initial searches, 326 articles were identified from electronic databases, and 201 articles remained after duplicates were removed. One hundred and forty-seven studies were excluded due to irrelevance after checking titles and abstracts. The remaining 54 studies were retrieved for full-text evaluation, and 36 studies were excluded due to the following reasons: affiliate study (*n* = 19), insufficient data (*n* = 9), and no appropriate control (*n* = 8). Reviewing the reference lists of relevant studies did not yield any new eligible studies. Eventually, 18 RCTs assessing 8207 DLBCL patients were collected in our study [22–39] (Fig. 1).

### Study characteristics

The baseline characteristics of the included studies are presented in Table 1. To summarize, the studies were published from 2002 to 2019, and 102–1418 patients were included in individual trials. Ten of the included studies were conducted in multiple countries, and the remaining eight studies were conducted in a single country. Mean age of included patients ranged from 47.0–69.5 years, and the disease status ranged from low to high risk. The quality of included studies was evaluated using the JADAD scale, and studies with a score of 4 or 5 were regarded as high quality. Overall, six studies scored 4, six studies scored 3, four studies scored 2, and the remaining two studies scored 1.

**Table 1:** Baseline characteristics of the studies included in this meta-analysis

Study	Country	Sample size	Mean age (years)	Men/women	Disease status	Stages	Intervention	Chemotherapy regimen	Study quality
Coiffier 2002 [22]	Multiple countries	399	69.0	199/200	Previously untreated DLBCL	I-IV	Rituximab (375 mg per square meter, on day 1 of each of the eight cycles of CHOP)	CHOP	4
Habermann 2006 [23]	Multiple countries	546	69.5	273/273	Previously untreated DLBCL	I-IV	Rituximab (375 mg per square meter, 7 and 3 days before cycle 1, and 2 days before cycles 3, 5, and, if administered, 7)	CHOP	3
Pfreundschuh 2006 [24]	Multiple countries	823	47.0	478/345	Good-prognosis DLBCL	I-IV	Rituximab (375 mg per square meter given IV on days 1, 22, 43, 64, 85, and 106 of the chemotherapy regimen)	CHOP	4
Avilés 2007 [25]	Mexico	196	59.8	105/91	High-risk DLBCL	III-IV	Rituximab (375 mg per square meter)	CEOP	2
Vellenga 2008 [26]	The Netherlands	225	54.5	130/95	Relapsed/progressive DLBCL	I-IV	Rituximab (375 mg per square meter was administered on day 5 of the DHAP course or on day 6 of the VIM course)	DHAP	1
Pfreundschuh 2008 [27]	Germany	1222	68.3	650/572	Previously untreated DLBCL	I-IV	Bi-weekly dosing of rituximab (375 mg per square meter)	CHOP	4
Avilés 2010 [28]	Mexico	100	50.2	48/52	Refractory DLBCL	III-IV	Rituximab (375 mg per square meter day 1 IV every cycle)	ESHAP	1
Vose 2013 [29]	US	224	57.7	142/82	Relapsed DLBCL	NA	Rituximab (375 mg per square meter on days 19 and 12) or I-tositumomab (dosimetric dose of 5 mCi on day 19 and therapeutic total-body dose of 0.75 Gy on day 12)	BEAM	4
Ketterer 2013 [30]	France	222	49.2	139/83	Localized low-risk DLBCL	I-II	Rituximab (375 mg per square meter was administered on days 1, 15, 29, and 43 of the regimen)	ACVBP	3
Seymour 2014 [31]	Multiple countries	787	61.0	387/400	Previously untreated DLBCL	I-III	Bevacizumab (10 mg kg^−1^ q2w or 15 mg kg^− 1^ q3w)	R-CHOP	2
Offner 2015 [32]	Multiple countries	164	59.0	88/76	Previously untreated DLBCL	I-IV	Bortezomib 1.3 mg per square meter by IV on days 1, 4, 8, and 11	R-CHOP	3
Fayad 2015 [33]	US	151	59.0	85/66	Relapsed DLBCL	I-IV	Dacetuzumab administered on days 1, 3, 8, and 15	R-ICE	3
Hu 2017 [34]	China	144	49.4	98/46	DLBCL	NA	Rituximab (375 mg per square meter was administered every 2 months for 1 year)	CHOP	2
van Imhoff 2017 [35]	Multiple countries	445	57.0	272/173	Relapsed or Refractory DLBCL	I-IV	Ofatumumab 1000 mg or rituximab 375 mg per square meter was administered for a total of four infusions (days 1 and 8 of cycle 1; day 1 of cycles 2 and 3 of DHAP)	DHAP	3
Vitolo 2017 [36]	Multiple countries	1418	62.0	752/666	Previously untreated advanced-stage DLBCL	I-IV	Obinutuzumab (1000 mg IV on days 1, 8, and 15 of cycle 1, and on day 1 of cycles 2 to 8) or rituximab (375 mg per square meter IV on day 1 of cycles 1 to 8)	CHOP	4
Czuczman 2017 [37]	Multiple countries	102	67.0	61/41	Relapsed or Refractory DLBCL	NA	lenalidomide (25 mg per day, 21 days of 28-day cycle) or rituximab (375 mg per square meter IV on days 1, 8, 15, and 22 of cycles 1 to 8)	GEO	2
Leonard 2017 [38]	Multiple countries	201	63.0	107/94	Previously untreated DLBCL	I-IV	Bortezomib 1.3 mg per square meter IV on days 1 and 4	R-CHOP	3
Younes 2009 [39]	Multiple countries	838	62.0	447/391	Previously untreated non-GCB DLBCL	I-IV	Ibrutinib (560 mg per day orally)	R-CHOP	4

### Overall survival

**Fig. 2:**
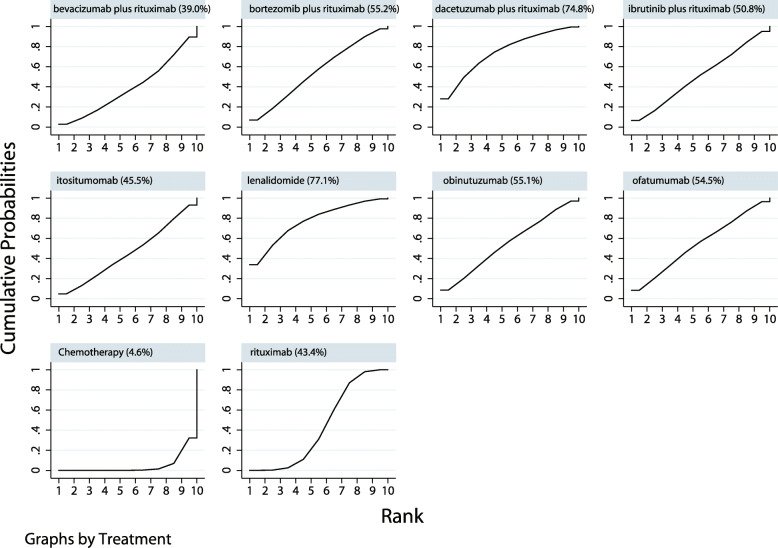
Cumulative ranking plots based on the estimated SUCRA probabilities for OS

The eligible comparisons of OS in the network plot including I-tositumomab, bevacizumab plus rituximab, bortezomib plus rituximab, dacetuzumab plus rituximab, ibrutinib plus rituximab, lenalidomide, obinutuzumab, ofatumumab, and rituximab treatments are presented in Supplemental 1. The nodes and the edges are weighted based on the number of studies in each treatment and on the precision of the direct pair-wise comparison, respectively. The SUCRA probabilities (%) were ranked to obtain comparative effects of these agents on OS, and the results indicated that the addition of dacetuzumab (74.8%) to rituximab-based regimens and lenalidomide (77.1%) was the most likely to improve OS (Fig. 2). The results of pair-wise comparisons agents are presented in Supplementals 2 and 3. Finally, no significant publication bias was detected through reviewing the funnel plot (Supplemental 4).

### Events-free survival

**Fig. 3:**
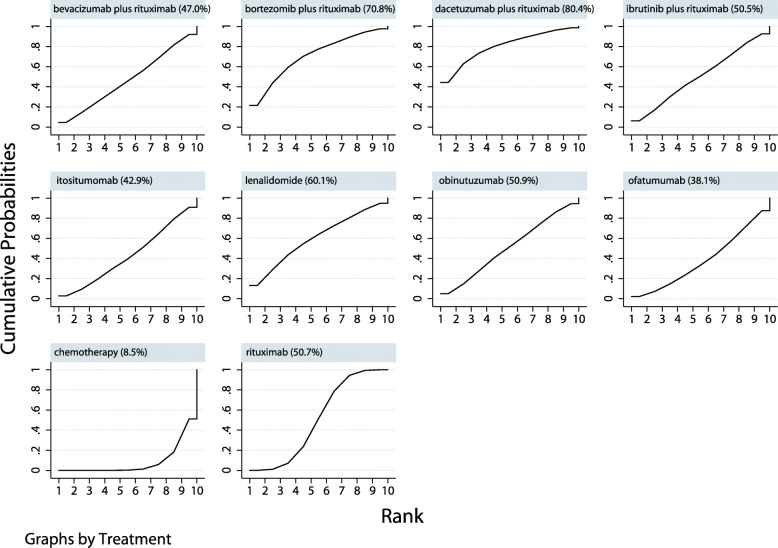
Cumulative ranking plots based on the estimated SUCRA probabilities for EFS

The eligible comparisons of EFS in the network plot including I-tositumomab, bevacizumab plus rituximab, bortezomib plus rituximab, dacetuzumab plus rituximab, ibrutinib plus rituximab, lenalidomide, obinutuzumab, ofatumumab, and rituximab are presented in Supplemental 1. The results of the SUCRA probabilities (%) indicated that dacetuzumab (80.4%) or bortezomib (70.8%) added to rituximab was the treatment most likely to improve EFS (Fig. 3). Supplementals 2 and 3 shows the details regarding the therapeutic effects of pair-wise comparisons agents on EFS. No significant publication bias was observed upon reviewing the funnel plot (Supplemental 4).

### Overall response rate

**Fig. 4:**
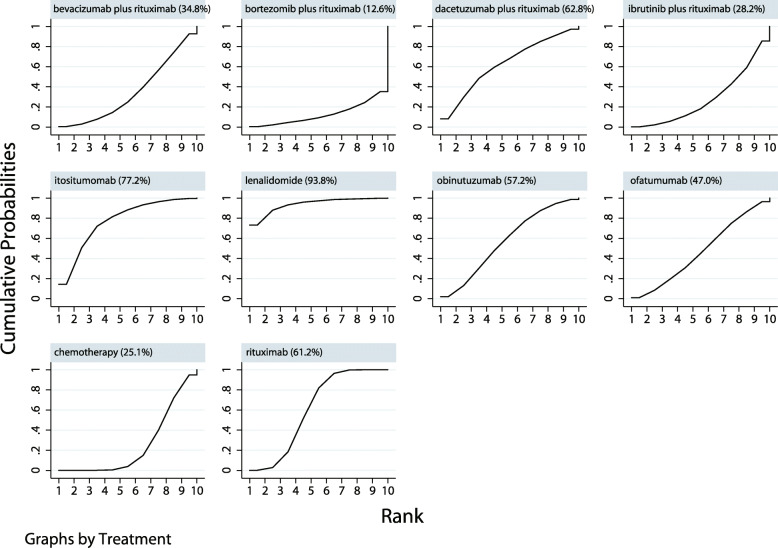
Cumulative ranking plots based on the estimated SUCRA probabilities for ORR

The network meta-analysis comparing the effects of various agents including I-tositumomab, bevacizumab plus rituximab, bortezomib plus rituximab, dacetuzumab plus rituximab, ibrutinib plus rituximab, lenalidomide, obinutuzumab, ofatumumab, and rituximab on ORR is presented in Supplemental 1. The SUCRA probabilities (%) indicated that lenalidomide (93.8%) produced the best therapeutic effect on ORR, and that I-tositumomab (77.2%) had a relatively good effect on ORR (Fig. 4). The results of pair-wise comparisons agents on ORR are listed in Supplementals 2 and 3. The funnel plot showed that there was no publication bias (Supplemental 4).

### Traditional meta-analysis

We firstly noted targeted therapies are significantly associated with improved OS, irrespective for previous untreated patients (HR: 0.82; 95%CI: 0.71–0.95; *P* = 0.008) or patients with relapsed or refractory DLBCL (HR: 0.85; 95%CI: 0.75–0.97; *P* = 0.016). Moreover, targeted therapies could significantly improved EFS for previous untreated patients (HR: 0.76; 95%CI: 0.63–0.92; *P* = 0.005), while it did not yield significant improvement in EFS for patients with relapsed or refractory DLBCL (HR: 0.77; 95%CI: 0.58–1.04; *P* = 0.084). Finally, targeted therapies have no significant effects on ORR, irrespective for previous untreated patients (RR: 1.00; 95%CI: 0.96–1.05; *P* = 0.869) or patients with relapsed or refractory DLBCL (RR: 1.11; 95%CI: 0.91–1.34; *P* = 0.302) (Table 2).

**Table 2:** Summary of the results for efficacy outcomes based on disease status

Outcomes	Group	HR or RR and 95%CI	*P* value	Heterogeneity (%)	*P* value for heterogeneity
OS	Previous untreated	0.82 (0.71–0.95)	0.008	29.9	0.180
	Relapsed or refractory	0.85 (0.75–0.97)	0.016	0.0	0.471
EFS	Previous untreated	0.76 (0.63–0.92)	0.005	72.8	< 0.001
	Relapsed or refractory	0.77 (0.58–1.04)	0.084	83.1	< 0.001
ORR	Previous untreated	1.00 (0.96–1.05)	0.869	73.5	< 0.001
	Relapsed or refractory	1.11 (0.91–1.34)	0.302	65.5	0.013

### Adverse events

The pooled results for the targeted therapies on the risk of grade 3 or greater adverse events are summarized in Table 3. Overall, we noted that targeted therapies were associated with an increased risk of diarrhea (RR: 2.63; 95%CI: 1.18–5.86; *P* = 0.019), and thrombocytopenia (RR: 1.48; 95%CI: 1.08–2.02; *P* = 0.015), whereas no significant differences were observed among groups for the risks of fever (*P* = 0.470), infection (*P* = 0.267), mucositis (*P* = 0.615), liver toxicity (*P* = 0.307), cardiac toxicity (*P* = 0.197), neurologic toxicity (*P* = 0.393), renal toxicity (*P* = 0.136), lung toxicity (*P* = 0.539), nausea or vomiting (*P* = 0.232), constipation (*P* = 0.560), neutropenia (*P* = 0.363), anemia (*P* = 0.096), leucocytopenia (*P* = 0.342), or granulocytopenia (*P* = 0.549).

**Table 3:** Summary of the results for grade 3 or greater adverse events

Outcomes	Number of cohorts	RR and 95%CI	*P* value	Heterogeneity (%)	*P* value for heterogeneity
Fever	3	0.83 (0.50–1.38)	0.470	8.6	0.335
Infection	9	1.14 (0.91–1.43)	0.267	62.9	0.006
Mucositis	4	1.15 (0.67–1.98)	0.615	48.6	0.120
Liver toxicity	3	0.63 (0.26–1.52)	0.307	0.0	0.481
Cardiac toxicity	8	1.31 (0.87–1.98)	0.197	18.0	0.288
Neurologic toxicity	9	0.88 (0.66–1.18)	0.393	0.0	0.572
Renal toxicity	3	0.36 (0.10–1.38)	0.136	0.0	0.773
Lung toxicity	7	1.16 (0.73–1.85)	0.539	33.8	0.170
Nausea or vomiting	7	0.75 (0.47–1.20)	0.232	0.0	0.481
Constipation	2	0.61 (0.12–3.20)	0.560	27.8	0.239
Diarrhea	4	2.63 (1.18–5.86)	0.019	0.0	0.569
Neutropenia	8	1.04 (0.95–1.14)	0.363	26.0	0.222
Anemia	11	1.18 (0.97–1.44)	0.096	21.4	0.239
Thrombocytopenia	11	1.48 (1.08–2.02)	0.015	54.9	0.014
Leucocytopenia	6	1.06 (0.94–1.20)	0.342	0.0	0.971
Granulocytopenia	2	1.16 (0.71–1.88)	0.549	23.7	0.252

## Discussion

The current network meta-analysis was carried out to compare the efficacy and safety of targeted therapies for DLBCL patients, and to investigate agents including I-tositumomab, bevacizumab plus rituximab, bortezomib plus rituximab, dacetuzumab plus rituximab, ibrutinib plus rituximab, lenalidomide, obinutuzumab, ofatumumab, and rituximab. This comprehensive quantitative study included 8207 DLBCL patients from 18 RCTs across a broad range of patient characteristics. The findings of this study indicated that the addition of dacetuzumab to rituximab-based regimens or lenalidomide was associated with greater improvements in OS, while dacetuzumab or bortezomib added to rituximab had a relatively good effect on EFS. Furthermore, DLBCL patients receiving lenalidomide or I-tositumomab experienced better ORR. Moreover, targeted therapies present an increased risk of diarrhea and thrombocytopenia compared with traditional chemotherapy or rituximab-based chemotherapy regimens.

This is the first meta-analysis to compare various targeted therapies for patients with DLBCL, whereas several other meta-analysis have provided the results for a single agent. A meta-analysis conducted by Lin et al. contained four studies and found that bortezomib-containing regimens did not yield significant improvements in survival outcomes, and might be associated with a greater risk of peripheral neuropathy compared to standard R-CHOP regimens [40]. Moreover, Ren et al. observed that rituximab salvage therapy was associated with better OS, PFS, and ORR for relapse or refractory DLBCL, whereas maintenance rituximab therapy did not significantly affect OS or EFS [41]. However, until now, no studies had compared the therapeutic effects of various agents for patients with DLBCL. Therefore, the current comprehensive network meta-analysis was conducted to elucidate the best treatment strategies for patients with DLBCL based on OS, EFS, and ORR.

The results of this study indicated that the addition of dacetuzumab to rituximab-based regimens or lenalidomide was associated with relatively good therapeutic effects on OS. However, the results of pair-wise comparisons indicated that dacetuzumab added to rituximab-based regimens or lenalidomide were associated with greater improvements in OS compared to chemotherapy, whereas no other significant differences between agents were observed. Moreover, dacetuzumab or bortezomib added to rituximab produced relatively good effects on EFS. One potential reason for this is that dacetuzumab directly affects malignant cells and antigen-presenting cells, especially dendritic cells [33]. Furthermore, DLBCL patients received lenalidomide or I-tositumomab experienced better therapeutic effects on ORR. Lenalidomide modulates CRL4^CRBN^ E3 ligase activity and the associating ubiquitination and subsequent proteasomal degradation of Aiolos and Ikaros, which could cause decreased proliferation of ABC-DLCBL cell lines and activation of immune cells such as T and natural killer cells [42, 43]. Although the results of pair-wise comparisons were mostly not statistically significant, these results could be due to the small number of studies.

The results of this study indicated that several treatments were associated with significant improvements in OS and EFS, without more severe adverse events occurring. However, the risk of diarrhea and thrombocytopenia in patients receiving targeted therapies was significantly increased. Moreover, the impact of the toxicity of targeted therapies on quality of life should be taken into account. However, a pooled conclusion on life quality was not drawn due to the fact that data on quality of life were rarely available, highlighting the need for further verification by large-scale RCTs.

There were several limitations in our study. First, the results of this study are at the study level, not at the individual level. Second, the characteristics of enrolled patients varied, which could have affected their prognoses. Third, stratified analyses according to study or patient characteristics were not conducted because several treatments were reported in a smaller number of trials. Forth, the background treatment strategies were not addressed, which could have biased survival outcomes. Finally, the analysis was based on published articles, and publication bias is inevitable.

## Conclusions

In conclusion, dacetuzumab to rituximab-based regimens or lenalidomide produces better effect on OS in DLBCL patients, while dacetuzumab or bortezomib added to rituximab is associated with greater improvements in EFS. Moreover, patients receiving lenalidomide or I-tositumomab have a relatively high ORR. Finally, patients receiving targeted therapies are at an increased risk of diarrhea and thrombocytopenia compared to those receiving traditional chemotherapy or rituximab-based chemotherapy regimens.

## Supplementary Information

**Additional file 1: Figure S1.** Network of comparisons for OS of included in the analyses. **Figure S2.** Network of comparisons for EFS of included in the analyses. **Figure S3.** Network of comparisons for ORR of included in the analyses.

**Additional file 2: Figure S1.** Pair-wise comparisons agents on OS. **Figure S2.** Pair-wise comparisons agents on EFS. **Figure S3.** Pair-wise comparisons agents on ORR.

**Additional file 3: Table S1.** Pair-wise comparisons agents on OS. **Table S2.** Pair-wise comparisons agents on EFS. **Table S3.** Pair-wise comparisons agents on ORR.

**Additional file 4: Figure S1.** Publication bias for OS. **Figure S2.** Publication bias for EFS. **Figure S3.** Publication bias for ORR.

## Data Availability

All data generated or analysed during this study are included in this published article [and its supplementary information files].

## Abbreviations

CI: Confidence intervals
DLBCL: Diffuse large B-cell lymphoma
EFS: Events-free survival
ORR: Overall response rate
OS: Overall survival
PRISMA: Preferred Reporting Items for Systematic Reviews and Meta-Analyses
RCTs: Randomized controlled trials
SUCRA: Surface under the cumulative ranking

## References

[1] Al-Hamadani M, Habermann TM, Cerhan JR, Macon WR, Maurer MJ, Go RS (2015). Non-Hodgkin lymphoma subtype distribution, geodemographic patterns, and survival in the US: a longitudinal analysis of the National Cancer Data Base from 1998 to 2011. Am J Hematol.

[2] International Non-Hodgkin’s Lymphoma Prognostic Factors P (1993). A predictive model for aggressive non-Hodgkin's lymphoma. N Engl J Med.

[3] Sehn LH, Berry B, Chhanabhai M, Fitzgerald C, Gill K, Hoskins P (2007). The revised international prognostic index (R-IPI) is a better predictor of outcome than the standard IPI for patients with diffuse large B-cell lymphoma treated with R-CHOP. Blood.

[4] Zhou Z, Sehn LH, Rademaker AW, Gordon LI, Lacasce AS, Crosby-Thompson A (2014). An enhanced international prognostic index (NCCN-IPI) for patients with diffuse large B-cell lymphoma treated in the rituximab era. Blood.

[5] Dunleavy K, Roschewski M, Wilson WH (2014). Precision treatment of distinct molecular subtypes of diffuse large B-cell lymphoma: ascribing treatment based on the molecular phenotype. Clin Cancer Res.

[6] Alizadeh AA, Eisen MB, Davis RE, Ma C, Lossos IS, Rosenwald A (2000). Distinct types of diffuse large B-cell lymphoma identified by gene expression profiling. Nature.

[7] Nowakowski GS, Czuczman MS (2015). ABC, GCB, and double-hit diffuse large B-cell lymphoma: does subtype make a difference in therapy selection?. Am Soc Clin Oncol Educ Book.

[8] Rosenwald A, Wright G, Chan WC, Connors JM, Campo E, Fisher RI (2002). The use of molecular profiling to predict survival after chemotherapy for diffuse large-B-cell lymphoma. N Engl J Med.

[9] Coiffier B, Thieblemont C, Van Den Neste E, Lepeu G, Plantier I, Castaigne S (2010). Long-term outcome of patients in the LNH-98.5 trial, the first randomized study comparing rituximab-CHOP to standard CHOP chemotherapy in DLBCL patients: a study by the Groupe d'Etudes des Lymphomes de l’Adulte. Blood.

[10] Pfreundschuh M, Kuhnt E, Trumper L, Osterborg A, Trneny M, Shepherd L (2011). CHOP-like chemotherapy with or without rituximab in young patients with good-prognosis diffuse large-B-cell lymphoma: 6-year results of an open-label randomised study of the MabThera international trial (MInT) group. Lancet Oncol.

[11] Sehn LH, Donaldson J, Chhanabhai M, Fitzgerald C, Gill K, Klasa R (2005). Introduction of combined CHOP plus rituximab therapy dramatically improved outcome of diffuse large B-cell lymphoma in British Columbia. J Clin Oncol.

[12] Moher D, Liberati A, Tetzlaff J, Altman DG (2009). Preferred reporting items for systematic reviews and meta-analyses: the PRISMA statement. PLoS Med.

[13] Jadad AR, Moore RA, Carroll D, Jenkinson C, Reynolds DJ, Gavaghan DJ (1996). Assessing the quality of reports of randomized clinical trials: is blinding necessary?. Control Clin Trials.

[14] White IR, Barrett JK, Jackson D, Higgins JP (2012). Consistency and inconsistency in network meta-analysis: model estimation using multivariate meta-regression. Res Synth Methods.

[15] Bucher HC, Guyatt GH, Griffith LE, Walter SD (1997). The results of direct and indirect treatment comparisons in meta-analysis of randomized controlled trials. J Clin Epidemiol.

[16] Li D, Wang T, Shen S, Cheng S, Yu J, Zhang Y (2015). Effects of Fluroquinolones in newly diagnosed, sputum-positive tuberculosis therapy: a systematic review and network meta-analysis. PLoS One.

[17] Trinquart L, Chatellier G, Ravaud P (2012). Adjustment for reporting bias in network meta-analysis of antidepressant trials. BMC Med Res Methodol.

[18] DerSimonian R, Laird N (1986). Meta-analysis in clinical trials. Control Clin Trials.

[19] Ades AE, Lu G, Higgins JP (2005). The interpretation of random-effects meta-analysis in decision models. Med Decis Mak.

[20] Deeks JJ, Higgins J, Altman DG. Analysing data and undertaking meta-analyses. In: Higgins J, Green S, editors. Cochrane handbook for systematic reviews of interventions. Oxford; 2008.

[21] Higgins JP, Thompson SG, Deeks JJ, Altman DG (2003). Measuring inconsistency in meta-analyses. BMJ (Clinical research ed).

[22] Coiffier B, Lepage E, Briere J, Herbrecht R, Tilly H, Bouabdallah R (2002). CHOP chemotherapy plus rituximab compared with CHOP alone in elderly patients with diffuse large-B-cell lymphoma. N Engl J Med.

[23] Habermann TM, Weller EA, Morrison VA, Gascoyne RD, Cassileth PA, Cohn JB (2006). Rituximab-CHOP versus CHOP alone or with maintenance rituximab in older patients with diffuse large B-cell lymphoma. J Clin Oncol.

[24] Pfreundschuh M, Trumper L, Osterborg A, Pettengell R, Trneny M, Imrie K (2006). CHOP-like chemotherapy plus rituximab versus CHOP-like chemotherapy alone in young patients with good-prognosis diffuse large-B-cell lymphoma: a randomised controlled trial by the MabThera international trial (MInT) group. Lancet Oncol.

[25] Aviles A, Nambo MJ, Neri N, Cleto S, Castaneda C, Huerta-Guzman J (2007). Dose dense (CEOP-14) vs dose dense and rituximab (CEOP-14 +R) in high-risk diffuse large cell lymphoma. Med Oncol (Northwood, London, England).

[26] Vellenga E, van Putten WL, van’t Veer MB, Zijlstra JM, Fibbe WE, van Oers MH (2008). Rituximab improves the treatment results of DHAP-VIM-DHAP and ASCT in relapsed/progressive aggressive CD20+ NHL: a prospective randomized HOVON trial. Blood.

[27] Pfreundschuh M, Schubert J, Ziepert M, Schmits R, Mohren M, Lengfelder E (2008). Six versus eight cycles of bi-weekly CHOP-14 with or without rituximab in elderly patients with aggressive CD20+ B-cell lymphomas: a randomised controlled trial (RICOVER-60). Lancet Oncol.

[28] Aviles A, Neri N, Huerta-Guzman J, de Jesus NM (2010). ESHAP versus rituximab-ESHAP in frail patients with refractory diffuse large B-cell lymphoma. Clin Lymphoma Myeloma Leuk.

[29] Vose JM, Carter S, Burns LJ, Ayala E, Press OW, Moskowitz CH (2013). Phase III randomized study of rituximab/carmustine, etoposide, cytarabine, and melphalan (BEAM) compared with iodine-131 tositumomab/BEAM with autologous hematopoietic cell transplantation for relapsed diffuse large B-cell lymphoma: results from the BMT CTN 0401 trial. J Clin Oncol.

[30] Ketterer N, Coiffier B, Thieblemont C, Ferme C, Briere J, Casasnovas O (2013). Phase III study of ACVBP versus ACVBP plus rituximab for patients with localized low-risk diffuse large B-cell lymphoma (LNH03-1B). Ann Oncol.

[31] Seymour JF, Pfreundschuh M, Trneny M, Sehn LH, Catalano J, Csinady E (2014). R-CHOP with or without bevacizumab in patients with previously untreated diffuse large B-cell lymphoma: final MAIN study outcomes. Haematologica.

[32] Offner F, Samoilova O, Osmanov E, Eom HS, Topp MS, Raposo J (2015). Frontline rituximab, cyclophosphamide, doxorubicin, and prednisone with bortezomib (VR-CAP) or vincristine (R-CHOP) for non-GCB DLBCL. Blood.

[33] Fayad L, Ansell SM, Advani R, Coiffier B, Stuart R, Bartlett NL (2015). Dacetuzumab plus rituximab, ifosfamide, carboplatin and etoposide as salvage therapy for patients with diffuse large B-cell lymphoma relapsing after rituximab, cyclophosphamide, doxorubicin, vincristine and prednisolone: a randomized, double-blind, placebo-controlled phase 2b trial. Leuk Lymphoma.

[34] Hu X, Zeng M, Yang SE, Liang X, Ding SS, Guo L (2017). Efficacy of rituximab combined with CHOP for treating patients with diffuse large B-cell lymphoma. Medicine.

[35] van Imhoff GW, McMillan A, Matasar MJ, Radford J, Ardeshna KM, Kuliczkowski K (2017). Ofatumumab versus rituximab salvage Chemoimmunotherapy in relapsed or refractory diffuse large B-cell lymphoma: the ORCHARRD study. J Clin Oncol.

[36] Vitolo U, Trneny M, Belada D, Burke JM, Carella AM, Chua N (2017). Obinutuzumab or rituximab plus cyclophosphamide, doxorubicin, vincristine, and prednisone in previously untreated diffuse large B-cell lymphoma. J Clin Oncol.

[37] Czuczman MS, Trneny M, Davies A, Rule S, Linton KM, Wagner-Johnston N (2017). A phase 2/3 multicenter, randomized, open-label study to compare the efficacy and safety of Lenalidomide versus Investigator's choice in patients with relapsed or refractory diffuse large B-cell lymphoma. Clin Cancer Res.

[38] Leonard JP, Kolibaba KS, Reeves JA, Tulpule A, Flinn IW, Kolevska T (2017). Randomized phase II study of R-CHOP with or without Bortezomib in previously untreated patients with non-germinal center B-cell-like diffuse large B-cell lymphoma. J Clin Oncol.

[39] Younes A, Sehn LH, Johnson P, Zinzani PL, Hong X, Zhu J (2019). Randomized phase III trial of Ibrutinib and rituximab plus cyclophosphamide, doxorubicin, vincristine, and prednisone in non-germinal center B-cell diffuse large B-cell lymphoma. J Clin Oncol.

[40] Lin Z, Chen X, Li Z, Zhou Y, Fang Z, Luo Y (2018). The role of bortezomib in newly diagnosed diffuse large B cell lymphoma: a meta-analysis. Ann Hematol.

[41] Ren YR, Jin YD, Zhang ZH, Li L, Wu P (2015). Rituximab treatment strategy for patients with diffuse large B-cell lymphoma after first-line therapy: a systematic review and meta-analysis. Chin Med J.

[42] Zhang LH, Kosek J, Wang M, Heise C, Schafer PH, Chopra R (2013). Lenalidomide efficacy in activated B-cell-like subtype diffuse large B-cell lymphoma is dependent upon IRF4 and cereblon expression. Br J Haematol.

[43] Gandhi AK, Kang J, Havens CG, Conklin T, Ning Y, Wu L (2014). Immunomodulatory agents lenalidomide and pomalidomide co-stimulate T cells by inducing degradation of T cell repressors Ikaros and Aiolos via modulation of the E3 ubiquitin ligase complex CRL4(CRBN.). Br J Haematol.

