# Supplementary material for: Network meta-analysis of targeted therapies for diffuse large B cell lymphoma
Source: BMC Cancer. 2020 Dec 11;20:1218. doi: 10.1186/s12885-020-07715-2 (PMC7733263; doi:10.1186/s12885-020-07715-2)
Supplement: Supplementary file 1 — Additional file 1: Figure S1. Network of comparisons for OS of included in the analyses. Figure S2. Network of comparisons for EFS of included in the analyses. Figure S3. Network of comparisons for ORR of included in the analyses. [file 12885_2020_7715_MOESM1_ESM.docx]

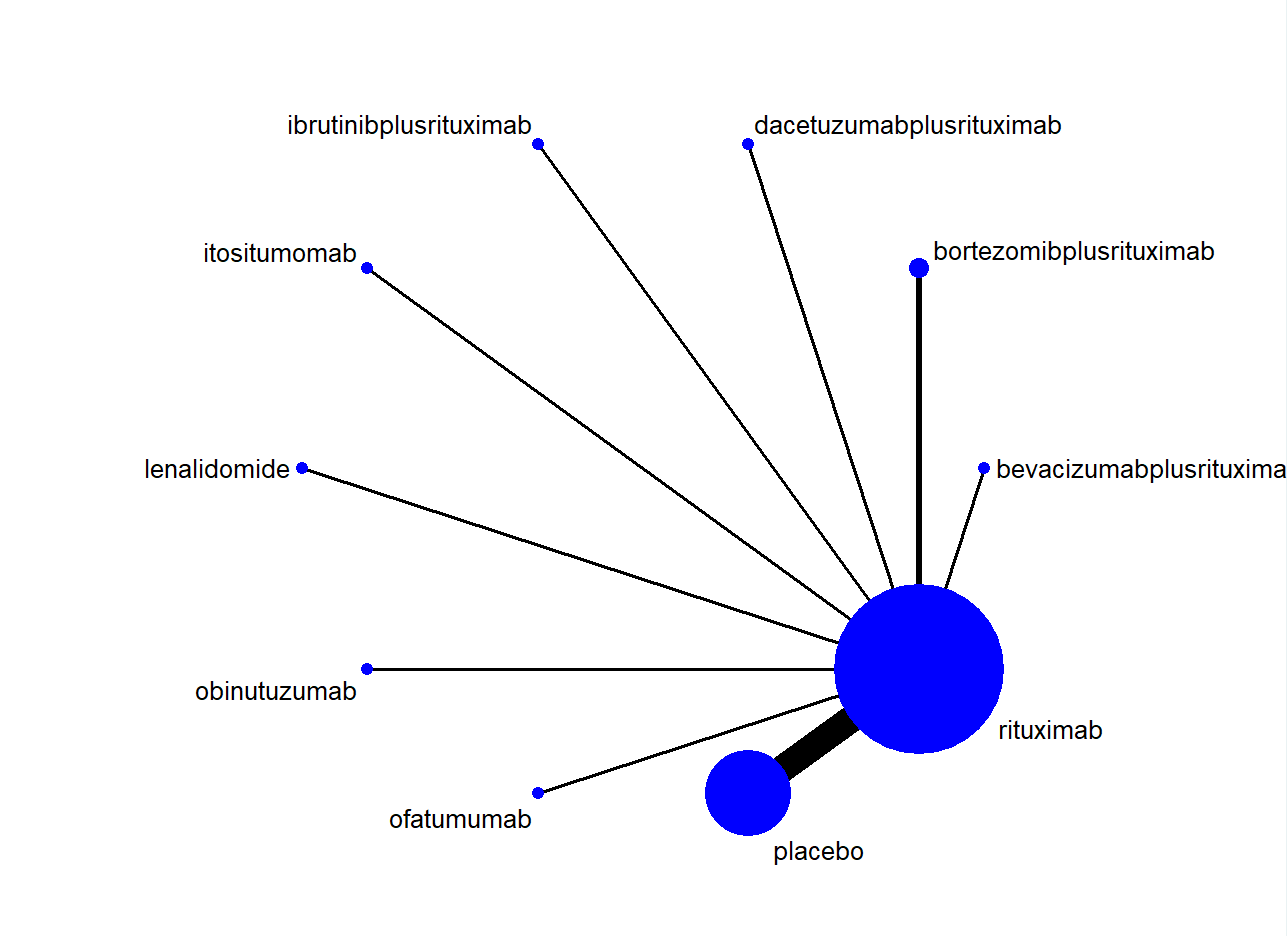


Figure S1. Network of comparisons for OS of included in the analyses


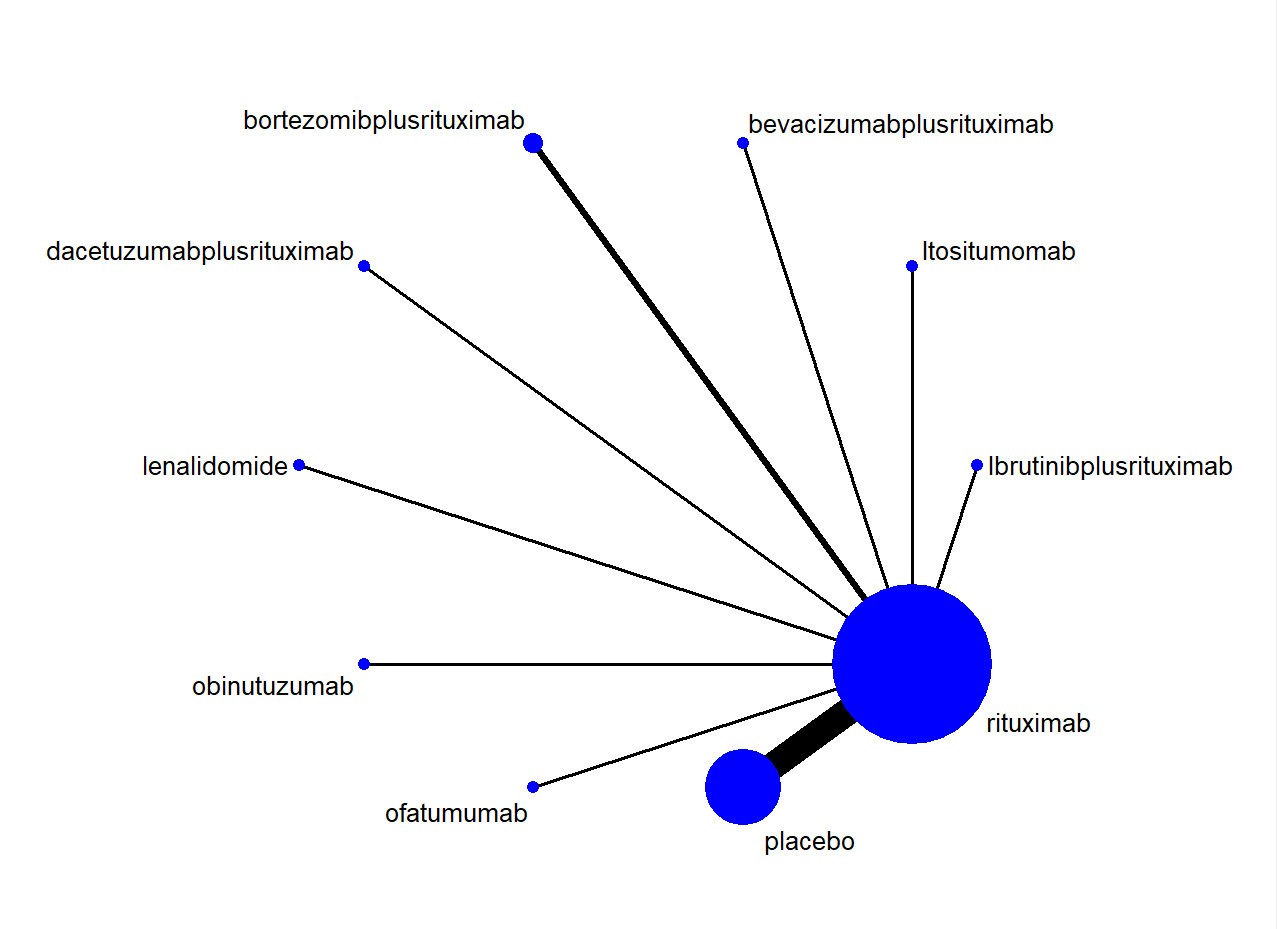


Figure S2. Network of comparisons for EFS of included in the analyses


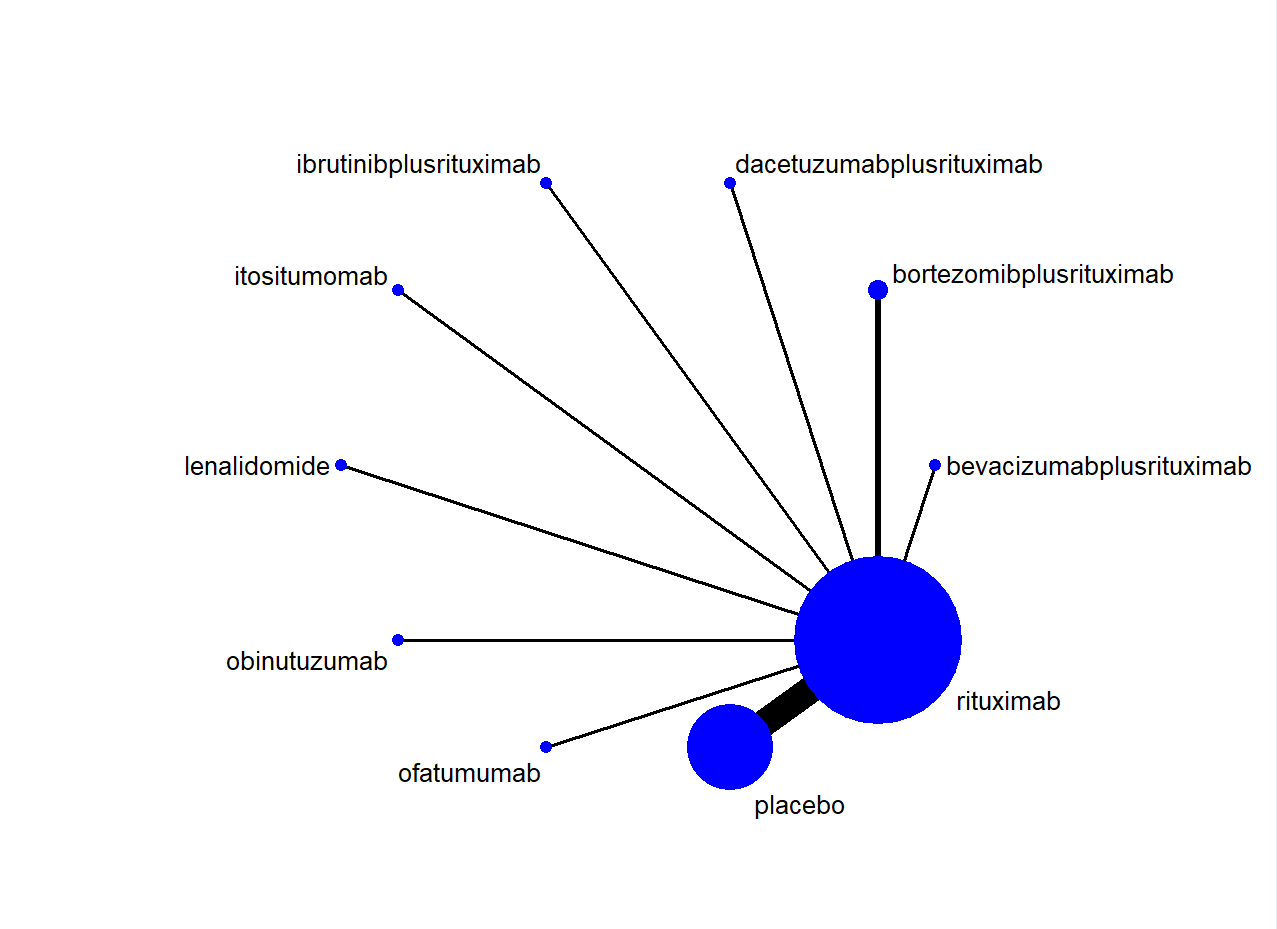


Figure S3. Network of comparisons for ORR of included in the analyses
