# Supplementary material for: Network meta-analysis of targeted therapies for diffuse large B cell lymphoma
Source: BMC Cancer. 2020 Dec 11;20:1218. doi: 10.1186/s12885-020-07715-2 (PMC7733263; doi:10.1186/s12885-020-07715-2)
Supplement: Supplementary file 2 — Additional file 2: Figure S1. Pair-wise comparisons agents on OS. Figure S2. Pair-wise comparisons agents on EFS. Figure S3. Pair-wise comparisons agents on ORR. [file 12885_2020_7715_MOESM2_ESM.docx]

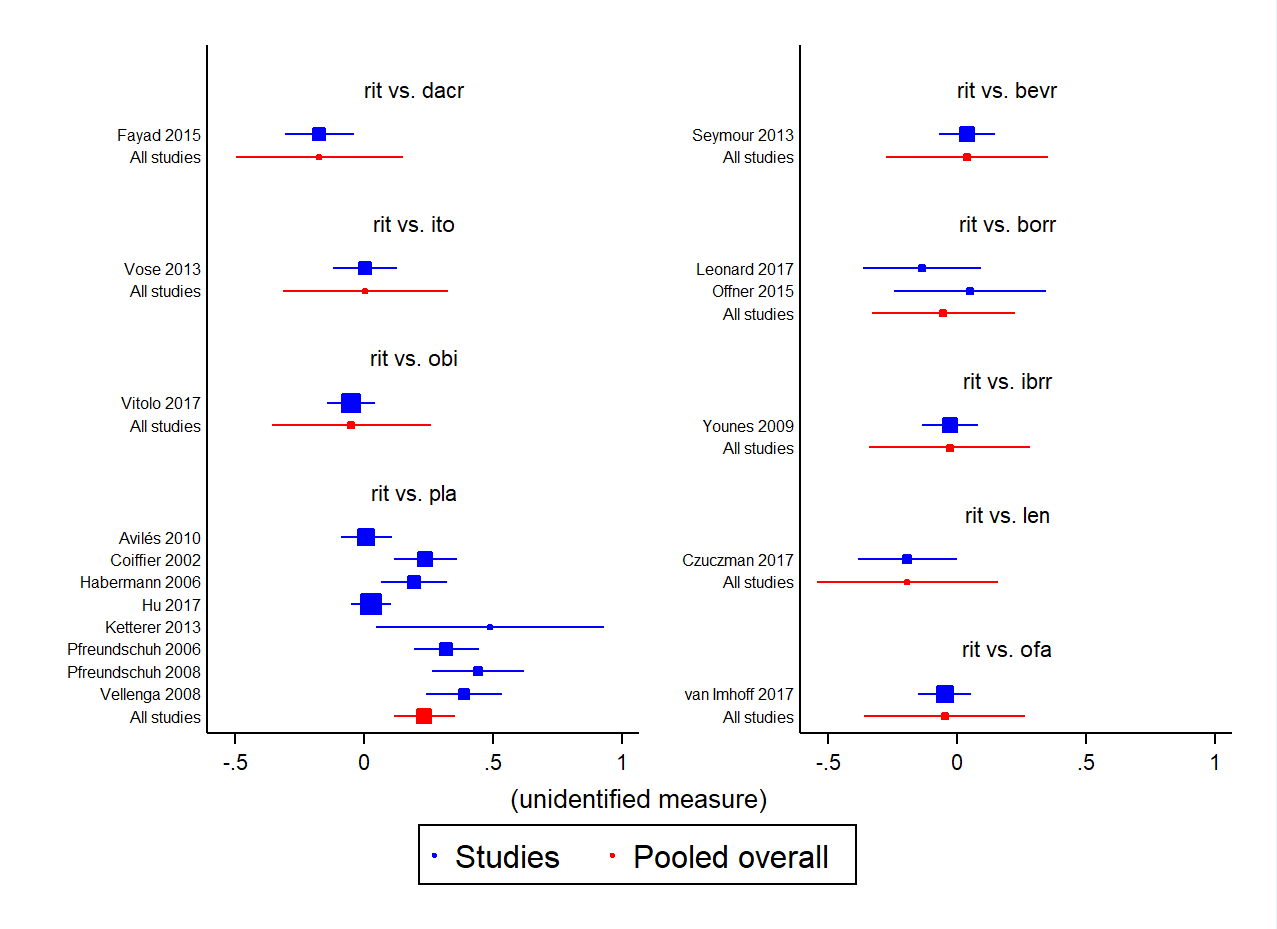


Figure S1. Pair-wise comparisons agents on OS


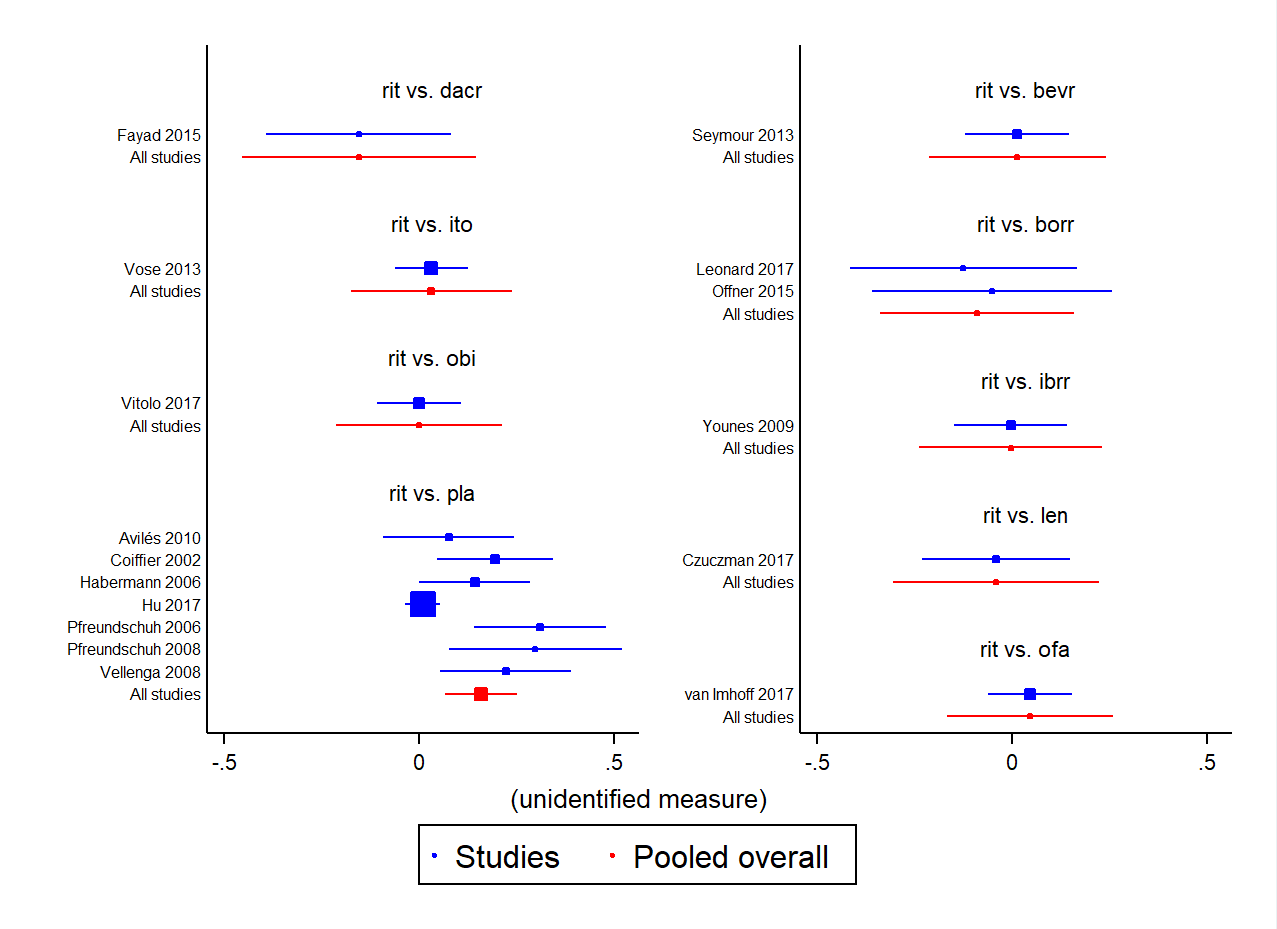


Figure S2. Pair-wise comparisons agents on EFS


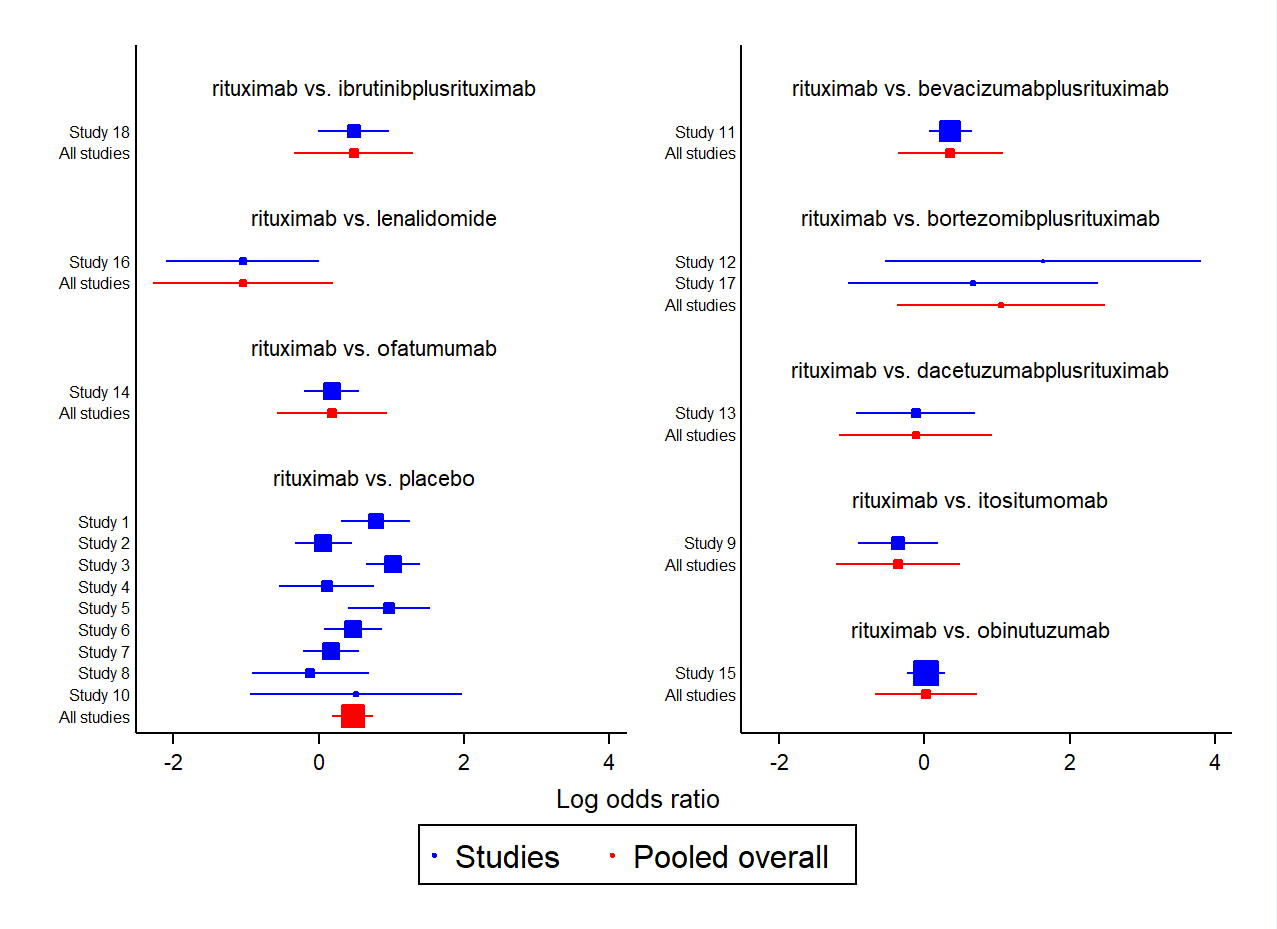


Figure S3. Pair-wise comparisons agents on ORR
