# Supplementary material for: Network meta-analysis of targeted therapies for diffuse large B cell lymphoma
Source: BMC Cancer. 2020 Dec 11;20:1218. doi: 10.1186/s12885-020-07715-2 (PMC7733263; doi:10.1186/s12885-020-07715-2)
Supplement: Supplementary file 3 — Additional file 3: Table S1. Pair-wise comparisons agents on OS. Table S2. Pair-wise comparisons agents on EFS. Table S3. Pair-wise comparisons agents on ORR. [file 12885_2020_7715_MOESM3_ESM.docx]

Table S1. Pair-wise comparisons agents on OS

| Comparator | bevr |  | borr |  | dacr |  | ibrr |  | ito |  | len |  | obi |  | ofa |  | pla |  | rit |  |
| --- | --- | --- | --- | --- | --- | --- | --- | --- | --- | --- | --- | --- | --- | --- | --- | --- | --- | --- | --- | --- |
| bevr |  |  | 1.1 | (0.72,1.66) | 1.24 | (0.79,1.94) | 1.07 | (0.69,1.67) | 1.03 | (0.66,1.62) | 1.26 | (0.79,2.02) | 1.09 | (0.70,1.69) | 1.09 | (0.70,1.70) | 0.82 | (0.59,1.15) | 1.04 | (0.76,1.42) |
| borr | 0.91 | (0.60,1.39) |  |  | 1.13 | (0.74,1.73) | 0.98 | (0.64,1.48) | 0.94 | (0.62,1.44) | 1.15 | (0.74,1.80) | 1 | (0.66,1.51) | 1 | (0.66,1.51) | 0.75 | (0.55,1.01) | 0.95 | (0.72,1.25) |
| dacr | 0.81 | (0.52,1.27) | 0.89 | (0.58,1.36) |  |  | 0.87 | (0.55,1.36) | 0.84 | (0.53,1.32) | 1.02 | (0.63,1.64) | 0.88 | (0.57,1.38) | 0.88 | (0.56,1.38) | 0.67 | (0.47,0.94) | 0.84 | (0.61,1.16) |
| ibrr | 0.94 | (0.60,1.46) | 1.02 | (0.67,1.56) | 1.16 | (0.74,1.81) |  |  | 0.97 | (0.62,1.51) | 1.18 | (0.74,1.89) | 1.02 | (0.66,1.58) | 1.02 | (0.66,1.59) | 0.77 | (0.55,1.07) | 0.97 | (0.71,1.33) |
| ito | 0.97 | (0.62,1.51) | 1.06 | (0.69,1.62) | 1.2 | (0.76,1.88) | 1.03 | (0.66,1.62) |  |  | 1.22 | (0.76,1.96) | 1.06 | (0.68,1.65) | 1.06 | (0.68,1.65) | 0.8 | (0.57,1.12) | 1 | (0.73,1.38) |
| len | 0.79 | (0.50,1.27) | 0.87 | (0.56,1.36) | 0.98 | (0.61,1.58) | 0.85 | (0.53,1.36) | 0.82 | (0.51,1.32) |  |  | 0.87 | (0.54,1.38) | 0.87 | (0.54,1.38) | 0.65 | (0.45,0.94) | 0.82 | (0.58,1.17) |
| obi | 0.92 | (0.59,1.42) | 1 | (0.66,1.52) | 1.13 | (0.72,1.77) | 0.98 | (0.63,1.52) | 0.95 | (0.61,1.47) | 1.15 | (0.72,1.84) |  |  | 1 | (0.64,1.55) | 0.75 | (0.54,1.05) | 0.95 | (0.70,1.29) |
| ofa | 0.92 | (0.59,1.43) | 1 | (0.66,1.52) | 1.13 | (0.72,1.77) | 0.98 | (0.63,1.53) | 0.95 | (0.61,1.48) | 1.16 | (0.72,1.85) | 1 | (0.65,1.55) |  |  | 0.75 | (0.54,1.05) | 0.95 | (0.70,1.30) |
| pla | 1.22 | (0.87,1.70) | 1.33 | (0.99,1.80) | 1.5 | (1.06,2.12) | 1.3 | (0.93,1.82) | 1.26 | (0.89,1.77) | 1.53 | (1.06,2.22) | 1.33 | (0.95,1.85) | 1.33 | (0.95,1.85) |  |  | 1.26 | (1.12,1.42) |
| rit | 0.96 | (0.70,1.32) | 1.06 | (0.80,1.39) | 1.19 | (0.86,1.64) | 1.03 | (0.75,1.41) | 1 | (0.72,1.37) | 1.21 | (0.85,1.72) | 1.05 | (0.77,1.43) | 1.05 | (0.77,1.43) | 0.79 | (0.70,0.89) |  |  |

Table S2. Pair-wise comparisons agents on EFS

| Comparator | beva |  | bort |  | dacr |  | ibrr |  | ito |  | len |  | obi |  | ofa |  | pla |  | rit |  |
| --- | --- | --- | --- | --- | --- | --- | --- | --- | --- | --- | --- | --- | --- | --- | --- | --- | --- | --- | --- | --- |
| beva |  |  | 1.11 | (0.79,1.55) | 1.18 | (0.81,1.72) | 1.02 | (0.73,1.41) | 0.98 | (0.72,1.33) | 1.06 | (0.75,1.49) | 1.01 | (0.74,1.38) | 0.97 | (0.71,1.32) | 0.86 | (0.68,1.10) | 1.01 | (0.81,1.27) |
| bort | 0.9 | (0.65,1.26) |  |  | 1.07 | (0.72,1.58) | 0.92 | (0.65,1.29) | 0.89 | (0.64,1.22) | 0.95 | (0.66,1.37) | 0.91 | (0.66,1.27) | 0.87 | (0.63,1.21) | 0.78 | (0.60,1.02) | 0.91 | (0.71,1.17) |
| dacr | 0.85 | (0.58,1.23) | 0.94 | (0.63,1.38) |  |  | 0.86 | (0.59,1.26) | 0.83 | (0.58,1.19) | 0.89 | (0.60,1.33) | 0.86 | (0.59,1.24) | 0.82 | (0.57,1.18) | 0.73 | (0.53,1.00) | 0.86 | (0.63,1.16) |
| ibrr | 0.98 | (0.71,1.36) | 1.09 | (0.77,1.53) | 1.16 | (0.80,1.70) |  |  | 0.97 | (0.71,1.32) | 1.04 | (0.73,1.48) | 1 | (0.73,1.37) | 0.95 | (0.69,1.31) | 0.85 | (0.66,1.09) | 1 | (0.79,1.26) |
| ito | 1.02 | (0.75,1.38) | 1.13 | (0.82,1.56) | 1.2 | (0.84,1.73) | 1.04 | (0.76,1.41) |  |  | 1.08 | (0.77,1.50) | 1.03 | (0.77,1.39) | 0.99 | (0.73,1.33) | 0.88 | (0.70,1.10) | 1.03 | (0.84,1.27) |
| len | 0.95 | (0.67,1.34) | 1.05 | (0.73,1.51) | 1.12 | (0.75,1.67) | 0.96 | (0.68,1.37) | 0.93 | (0.67,1.30) |  |  | 0.96 | (0.68,1.35) | 0.92 | (0.65,1.29) | 0.82 | (0.62,1.08) | 0.96 | (0.74,1.25) |
| obi | 0.99 | (0.72,1.35) | 1.09 | (0.79,1.52) | 1.17 | (0.81,1.69) | 1 | (0.73,1.38) | 0.97 | (0.72,1.30) | 1.04 | (0.74,1.46) |  |  | 0.96 | (0.71,1.29) | 0.85 | (0.68,1.08) | 1 | (0.81,1.24) |
| ofa | 1.03 | (0.76,1.41) | 1.14 | (0.83,1.59) | 1.22 | (0.85,1.77) | 1.05 | (0.77,1.44) | 1.01 | (0.75,1.36) | 1.09 | (0.78,1.53) | 1.05 | (0.77,1.41) |  |  | 0.89 | (0.71,1.13) | 1.05 | (0.85,1.30) |
| pla | 1.16 | (0.91,1.48) | 1.28 | (0.98,1.67) | 1.37 | (1.00,1.87) | 1.18 | (0.91,1.51) | 1.13 | (0.91,1.42) | 1.22 | (0.92,1.61) | 1.17 | (0.93,1.48) | 1.12 | (0.89,1.41) |  |  | 1.17 | (1.07,1.28) |
| rit | 0.99 | (0.79,1.24) | 1.09 | (0.85,1.40) | 1.17 | (0.87,1.58) | 1 | (0.79,1.27) | 0.97 | (0.79,1.19) | 1.04 | (0.80,1.36) | 1 | (0.81,1.24) | 0.96 | (0.77,1.18) | 0.85 | (0.78,0.94) |  |  |

Table S3. Pair-wise comparisons agents on ORR

| _bevr_ | _borr_ | _dacr_ | _ibrr_ | _ito_ | _len_ | _obi_ | _ofa_ | _pla_ | _rit_ |  |  |  |  |  |  |  |  |  |
| --- | --- | --- | --- | --- | --- | --- | --- | --- | --- | --- | --- | --- | --- | --- | --- | --- | --- | --- |
| bevr | 0.5 | (0.10,2.48) | 1.61 | (0.45,5.75) | 0.89 | (0.30,2.63) | 2.06 | (0.68,6.29) | 4.07 | (0.97,17.02) | 1.4 | (0.51,3.81) | 1.2 | (0.42,3.40) | 0.9 | (0.42,1.95) | 1.43 | (0.70,2.94) |
| 2 | (0.40,9.92) | borr | 3.23 | (0.55,19.02) | 1.77 | (0.34,9.20) | 4.13 | (0.78,21.82) | 8.15 | (1.23,53.98) | 2.79 | (0.57,13.75) | 2.4 | (0.48,12.09) | 1.8 | (0.42,7.74) | 2.87 | (0.69,12.00) |
| 0.62 | (0.17,2.21) | 0.31 | (0.05,1.82) | dacr | 0.55 | (0.15,2.07) | 1.28 | (0.33,4.93) | 2.52 | (0.50,12.76) | 0.87 | (0.25,3.05) | 0.74 | (0.20,2.70) | 0.56 | (0.19,1.65) | 0.89 | (0.31,2.54) |
| 1.13 | (0.38,3.34) | 0.56 | (0.11,2.92) | 1.82 | (0.48,6.87) | ibrr | 2.33 | (0.71,7.57) | 4.59 | (1.04,20.19) | 1.58 | (0.54,4.62) | 1.35 | (0.45,4.10) | 1.01 | (0.43,2.40) | 1.62 | (0.72,3.65) |
| 0.49 | (0.16,1.48) | 0.24 | (0.05,1.28) | 0.78 | (0.20,3.02) | 0.43 | (0.13,1.40) | ito | 1.97 | (0.44,8.87) | 0.68 | (0.22,2.04) | 0.58 | (0.19,1.82) | 0.44 | (0.18,1.07) | 0.7 | (0.30,1.63) |
| 0.25 | (0.06,1.03) | 0.12 | (0.02,0.81) | 0.4 | (0.08,2.01) | 0.22 | (0.05,0.96) | 0.51 | (0.11,2.28) | len | 0.34 | (0.08,1.42) | 0.29 | (0.07,1.25) | 0.22 | (0.06,0.79) | 0.35 | (0.10,1.21) |
| 0.72 | (0.26,1.95) | 0.36 | (0.07,1.76) | 1.16 | (0.33,4.08) | 0.63 | (0.22,1.86) | 1.48 | (0.49,4.46) | 2.92 | (0.70,12.08) | obi | 0.86 | (0.31,2.41) | 0.64 | (0.30,1.37) | 1.03 | (0.51,2.07) |
| 0.83 | (0.29,2.37) | 0.42 | (0.08,2.10) | 1.35 | (0.37,4.90) | 0.74 | (0.24,2.25) | 1.72 | (0.55,5.38) | 3.4 | (0.80,14.47) | 1.17 | (0.42,3.27) | ofa | 0.75 | (0.33,1.68) | 1.2 | (0.56,2.55) |
| 1.11 | (0.51,2.41) | 0.56 | (0.13,2.39) | 1.8 | (0.61,5.32) | 0.99 | (0.42,2.34) | 2.29 | (0.93,5.64) | 4.53 | (1.27,16.11) | 1.55 | (0.73,3.31) | 1.33 | (0.59,2.99) | pla | 1.6 | (1.20,2.12) |
| 0.7 | (0.34,1.43) | 0.35 | (0.08,1.46) | 1.12 | (0.39,3.21) | 0.62 | (0.27,1.40) | 1.44 | (0.61,3.37) | 2.84 | (0.82,9.78) | 0.97 | (0.48,1.96) | 0.84 | (0.39,1.78) | 0.63 | (0.47,0.83) | rit |
