# Supplementary material for: Network meta-analysis of targeted therapies for diffuse large B cell lymphoma
Source: BMC Cancer. 2020 Dec 11;20:1218. doi: 10.1186/s12885-020-07715-2 (PMC7733263; doi:10.1186/s12885-020-07715-2)
Supplement: Supplementary file 4 — Additional file 4: Figure S1. Publication bias for OS. Figure S2. Publication bias for EFS. Figure S3. Publication bias for ORR. [file 12885_2020_7715_MOESM4_ESM.docx]

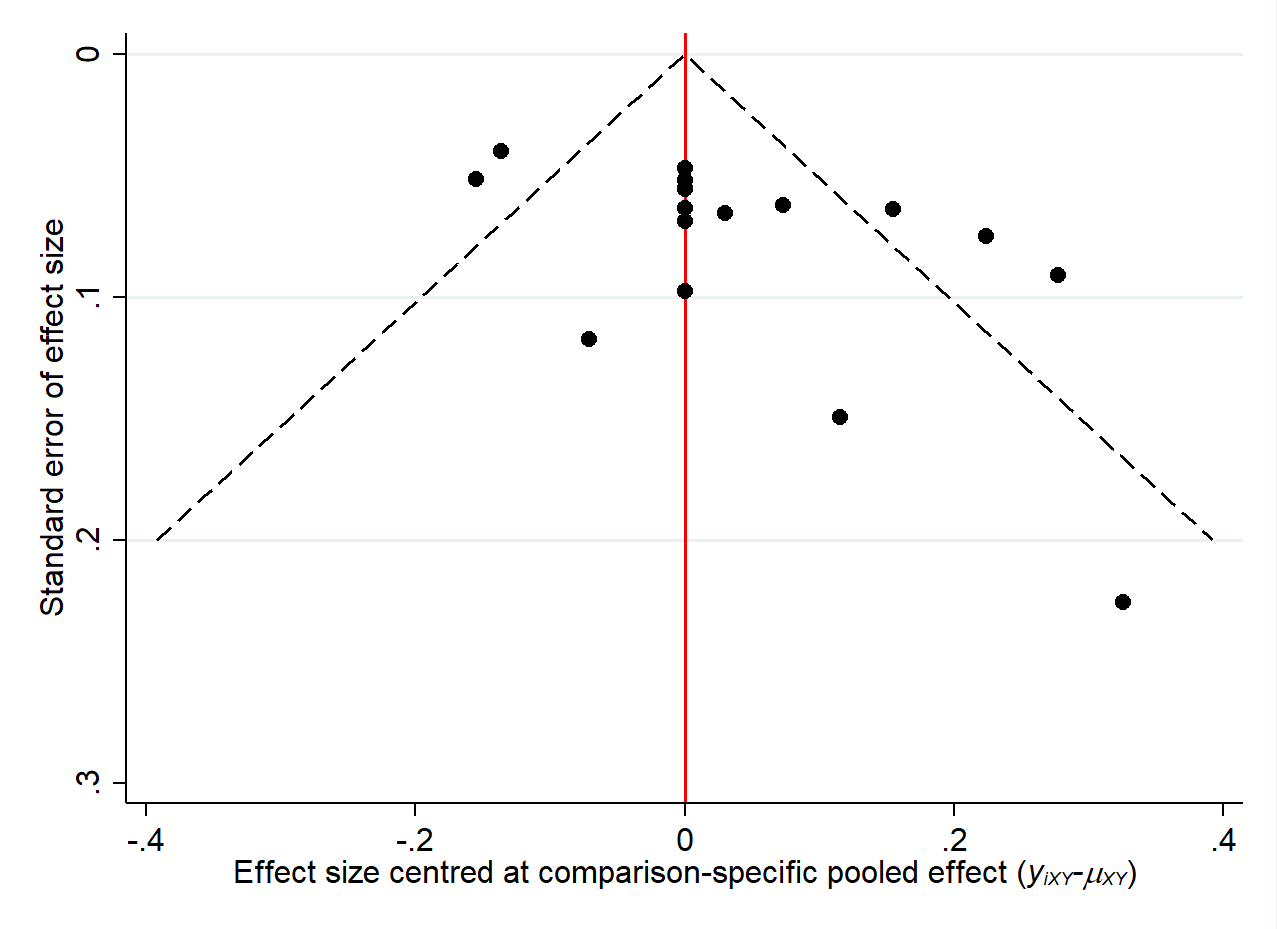


Figure S1. Publication bias for OS


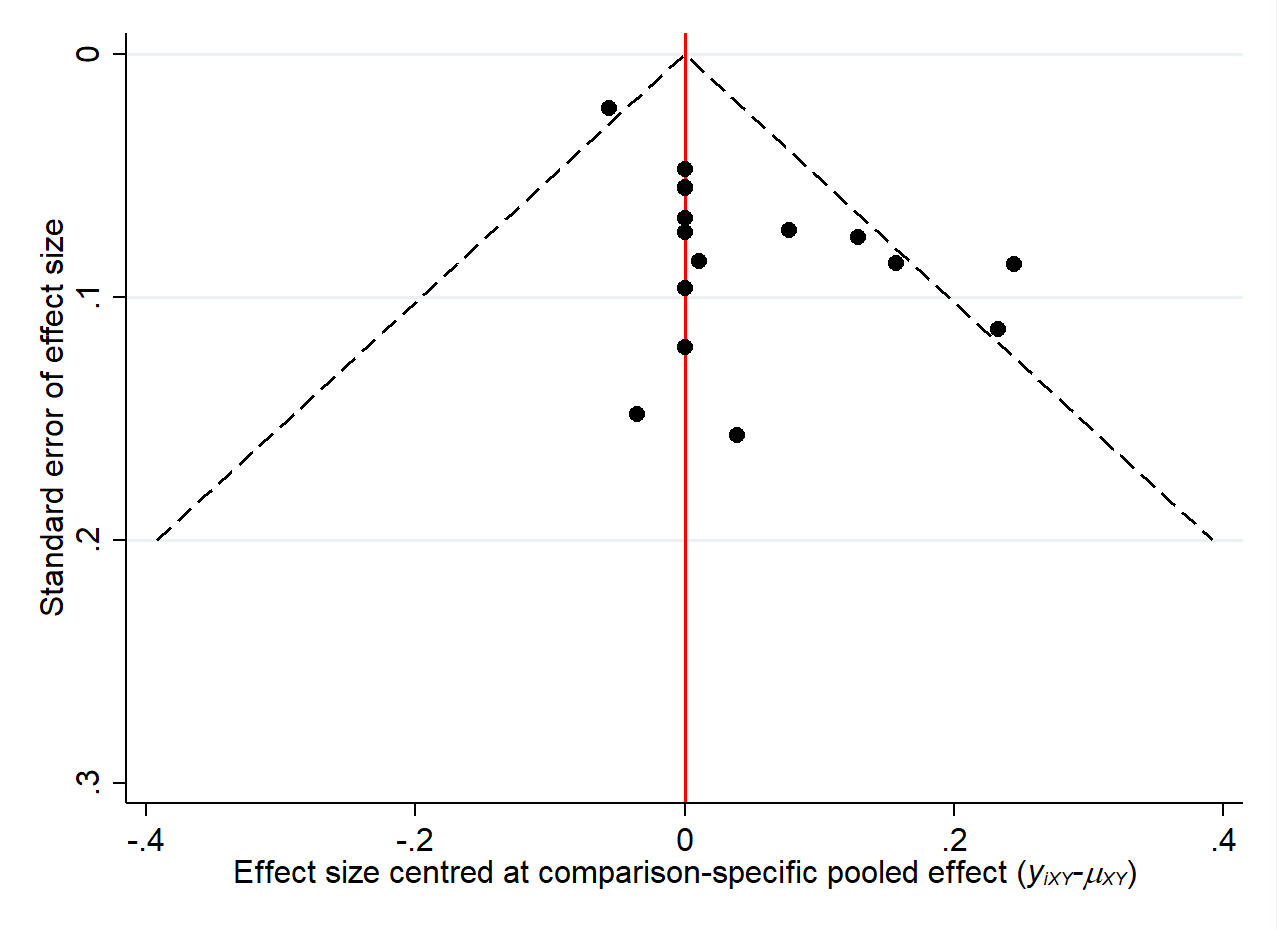


Figure S2. Publication bias for EFS


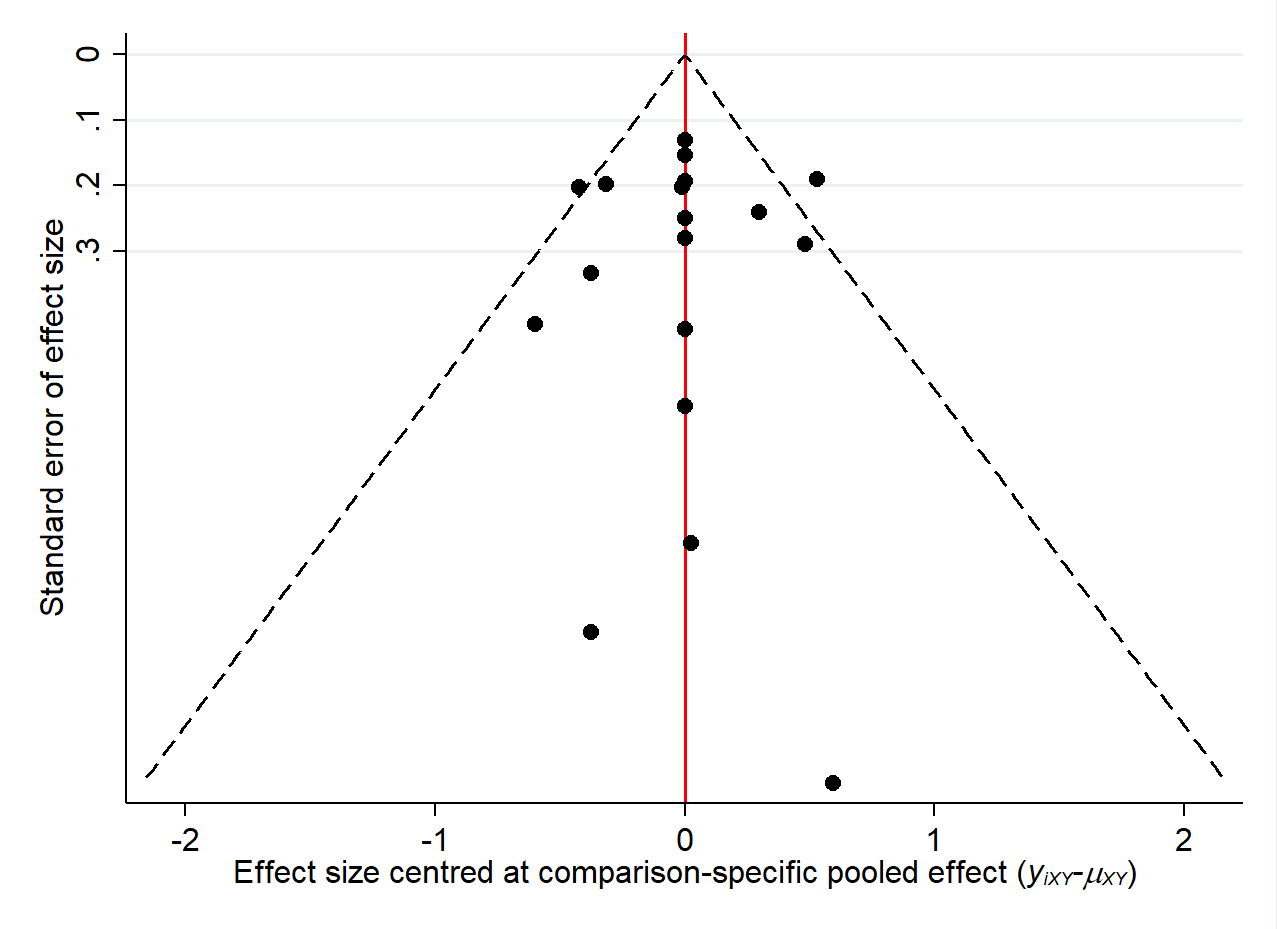


Figure S3. Publication bias for ORR
